# Possible kidney-lung cross-talk in COVID-19: in silico modeling of SARS-CoV-2 infection

**DOI:** 10.1186/s12882-022-02682-1

**Published:** 2022-02-05

**Authors:** Dmitry N. Grigoryev, Hamid Rabb

**Affiliations:** 1 Center for Translational Data Science, University of Chicago Division of Biological Sciences, 5454 South Shore Drive, Suite 2A/B, Chicago, IL 60615 USA; 2 Johns Hopkins Medicine, Baltimore, MD USA

**Keywords:** Acute kidney injury, Kidney-lung cross-talk, COVID receptors, ACE2, TMPRSS2, Genomics, Microarray, Gene expression

## Abstract

**Background:**

Publicly available genomics datasets have grown drastically during the past decades. Although most of these datasets were initially generated to answer a pre-defined scientific question, their repurposing can be useful when new challenges such as COVID-19 arise. While the establishment and use of experimental models of COVID-19 are in progress, the potential hypotheses for mechanisms of onset and progression of COVID-19 can be generated by using in silico analysis of known molecular changes during COVID-19 and targets for SARS-CoV-2 invasion.

**Methods:**

Selecting condition: COVID-19 infection leads to pneumonia and mechanical ventilation (PMV) and associated with acute kidney injury (AKI). There is increasing data demonstrating mechanistic links between AKI and lung injury caused by mechanical ventilation.

Selecting targets: SARS-CoV-2 uses angiotensin-converting enzyme 2 (ACE2) and transmembrane protease serine 2 (TMPRSS2) for cell entry. We hypothesized that expression of ACE2 and TMPRSS2 would be affected in models of AKI and PMV. We therefore evaluated expression of ACE2 and TMPRSS2 as well as other novel molecular players of AKI and AKI-lung cross-talk in the publicly available microarray datasets GSE6730 and GSE60088, which represent gene expression of lungs and kidneys in mouse models of AKI and PMV, respectively.

**Results:**

Expression of COVID-19 related genes ACE2 and TMPRSS2 was downregulated in lungs after 6 h of distant AKI effects. The expression of ACE2 decreased further after 36 h, while expression of TMPRSS2 recovered. In kidneys, both genes were downregulated by AKI, but not by distant lung injury. We also identified 53 kidney genes upregulated by PMV; and 254 lung genes upregulated by AKI, 9 genes of which were common to both organs. 3 of 9 genes were previously linked to kidney-lung cross-talk: Lcn2 (Fold Change (FC)_Lung (L)_ = 18.6, FC_Kidney (K)_ = 6.32), Socs3 (FC_L_ = 10.5, FC_K_ = 10.4), Inhbb (FC_L_ = 6.20, FC_K_ = 6.17). This finding validates the current approach and reveals 6 new candidates, including Maff (FC_L_ = 7.21, FC_K_ = 5.98).

**Conclusions:**

Using our in silico approach, we identified changes in COVID-19 related genes ACE2 and TMPRSS2 in traditional mouse models of AKI and kidney-lung cross-talk. We also found changes in new candidate genes, which could be involved in the combined kidney-lung injury during COVID-19.

## Background

Publicly available genomics datasets have grown drastically during the past decades. Although most datasets were initially generated to answer a pre-defined scientific question, the enormous amount of accumulated data makes genomics data repurposing possible when new challenges such as COVID-19 arise and need to be rapidly dealt with. These data sets are also very useful to study when traditional wet-lab research is slowed down during the pandemic, and experimental models of COVID-19 are still under development.

Clinical data for COVID-19 suggested that its mortality and morbidity can be linked not only to respiratory complications but also to other organ dysfunction [1]. Acute kidney injury (AKI) is associated with COVID-19. This association is thought to be secondary to hemodynamic effects of the disease related septic condition, and the poor outcome is secondary to sepsis, where AKI is often a surrogate marker of severity of illness in these patients [2, 3]. There is also a strong association between AKI and dysfunction of extra-renal organs, and more recent animal research has shown a significant causal effect of AKI on a distant organ dysfunction [4–8]. Despite frustrating outcomes, little is known about the potential pathophysiological interactions between the kidney and the extrarenal organs in critically ill patients. The critical illness during COVID-19 warrants study COVID-19 related genes and kidney-lung cross-talk to discover potential therapeutic targets.

We previously used combined genomics data analysis of kidney and lung to discover new molecular pathways of kidney-lung cross-talk during lung injury [9], and demonstrated that inflammation is a major component of the initiation and exacerbation of AKI. To relate our findings to COVID-19, we re-visited the global gene expression profiles of kidney in mice with pneumonia and mechanical ventilation [10, 11] with the main focus on changes in two important COVID-19 related genes angiotensin-converting enzyme 2 (ACE2) and transmembrane protease serine 2 (TMPRSS2). These genes were not previously considered during prior studies of kidney-lung cross-talk. However, SARS-CoV-2 uses ACE2 to enter both kidney and lung, and is highly expressed in proximal tubule cells of the kidney [10]. While virus enters the lung directly by inhalation the kidney becomes infected via blood (viremia), that occurs in ~ 15% of patients and is reported in association with severe infection [12]. The rate of coronoviral infection is multiplied by thousand times in the presence of co-expressed proteases [13]. TMPRSS2 gene codes for such a protease, which cleaves (primes) S protein of SARS-CoV-2 bound to ACE2 receptor [12]. TMPRSS2 is abundantly expressed in the distal nephron [14]. The data from Human Protein Atlas (https://www.proteinatlas.org) also demonstrates high co-expression of ACE2 and TMPRSS2 in microvilli of proximal tubules.

In addition to detected significant changes in expression of ACE2 and TMPRSS2 in AKI and PMV models, we also identified novel candidates that mediate cross-talk between the lung and kidney, which could be pertinent to COVID-19.

## Methods

### Study aims

This expression data repurposing study aims to extrapolate the molecular changes during COVID-19 to cross-talk between the injured kidney and lung using publicly available genomic data. Mouse models were selected based upon the phenotypes similar to those in patients with COVID-19. Two major COVID-19 related clinical conditions were selected: PMV and AKI. We hypothesized that genes involved in SARS-CoV-2 invasion will be affected in these models and might be potentially involved in COVID-19 outcome.

The microarray Genomic Series GSE60088 and GSE6730 were downloaded from the Gene Expression Omnibus (GEO).

Brief description of gene expression series 60,088: The *S. aureus* (SA) ~ 10^7^ cfu was deposited in mice oropharynx (*n* = 5) and mice were intubated via tracheostomy with a 20-gauge blunt metal catheter. Although the injurious pathogen in the model was bacterium the recent clinical observations showed that 37% of critically ill COVID-19 patients acquired bacterial pneumonia [15], which supports the model selection. Intubated mice were connected to a MiniVent rodent ventilator and mechanically ventilated (MV) with a tidal volume of 10 mL/kg, a respiratory rate of 150 breaths per minute. Control mice (*n* = 5) were maintained in their cages. After 6 h the lungs and kidneys were collected for RNA isolation. The Office of Animal Welfare at the University of Washington approved all experiments.

Brief description of gene expression series 6730: Male 6–8-week-old mice (C57BL6/J), weighing approximately 25-30 g were placed on a heating blanket and underwent midline laparotomy with isolation of bilateral renal pedicles. For mice assigned to experimental ischemia-reperfusion injury (IRI), a non-traumatic microvascular clamp was applied across both renal pedicles for 60 min. After the allotted ischemia time, the clamps were gently removed, the animals were administered 1 ml of sterile saline intraperitoneally, and the incision was closed in two layers with 4-0 silk suture. The animals were then allowed to recover with free access to food and water. Sham animals underwent the identical procedure without placement of the vascular clamps. At 6 or 36 h following the experimental procedure, the mice were euthanized by exsanguination under pentobarbital anesthesia and lung tissues were collected for the analysis. All procedures were approved by the Johns Hopkins Animal Care and Use Committee, and were consistent with the National Institutes of Health (NIH) Guide for the Care and Use of Laboratory Animals.

Mouse Genome 430 2.0 microarray (Affymetrix) was used for both models.

### Statistical analysis

Differentially expressed genes were identified using GEO-build-in GEO2R tool. For PMV model the groups were defined as follow: Control Lung Genomic Sample GSM1464822-GSM1464825, MV + SA Lung GSM1464826-GSM14648230. Control Kidney GSM1464839-GSM1464843, MV + SA Kidney GSM1464844-GSM1464848.

For AKI model the groups were defined as follow: Control 36 h GSM155092- GSM155096, AKI 36 h GSM155100- GSM155102. Control 6 h GSM155086- GSM155088, AKI 6 h GSM155089- GSM155091. Then GEO2R outputs comparing all groups were downloaded.

The main SARS-CoV-2 targets: angiotensin-converting enzyme 2 (ACE2) and transmembrane protease serine 2 (TMPRSS2) were selected for the detailed expression analysis.

## Results

We identified changes in lungs and kidneys of COVID-19 related genes ACE2 and TMPRSS2 in pneumonia and mechanical ventilation (PMV) and ischemic AKI-induced lung dysfunction (Table 1). Expression of ACE2 in lungs was significantly downregulated after 6 h in PMV model, and was similar to the gene downregulation in initially normal lungs at 6 h after the induction of ischemic injury to kidneys in AKI model. Lung ACE2 was further downregulated 36 h after ischemic AKI. (Table 1). Expression of TMPRSS2 in the lung was also significantly decreased after PMV, as it was 6 h after ischemic AKI. However, at 36 h of AKI lung TMPRSS2 was similar to the baseline.

**Table 1:** Response of ACE2 and TMPRSS2 to direct injurious stimuli in kidney and lung

		ACE2		TMPRSS2	
**Organ**	**Condition**	**FC**	***P*-value**	**FC**	***P*-value**
**LUNG**	PMV	−1.71	0.00037	−1.69	0.00002
	AKI 6 h	−1.86	0.00116	−1.43	0.00022
	AKI 36 h	−2.56	0.00062	−1.27	NS
**KIDNEY**	PMV	1.05	NS	1.17	NS
	AKI 6 h^a^	−2.44	0.00108	−1.76	0.01224
	AKI 36 h^a^	−3.27	0.00033	1.03	NS

^a^ the data was extracted from supplemental materials Grigoryev et al [9]

*AKI* acute kidney injury, *PMV* pneumonia and mechanical ventilation, *FC* fold change

**Fig. 1:**
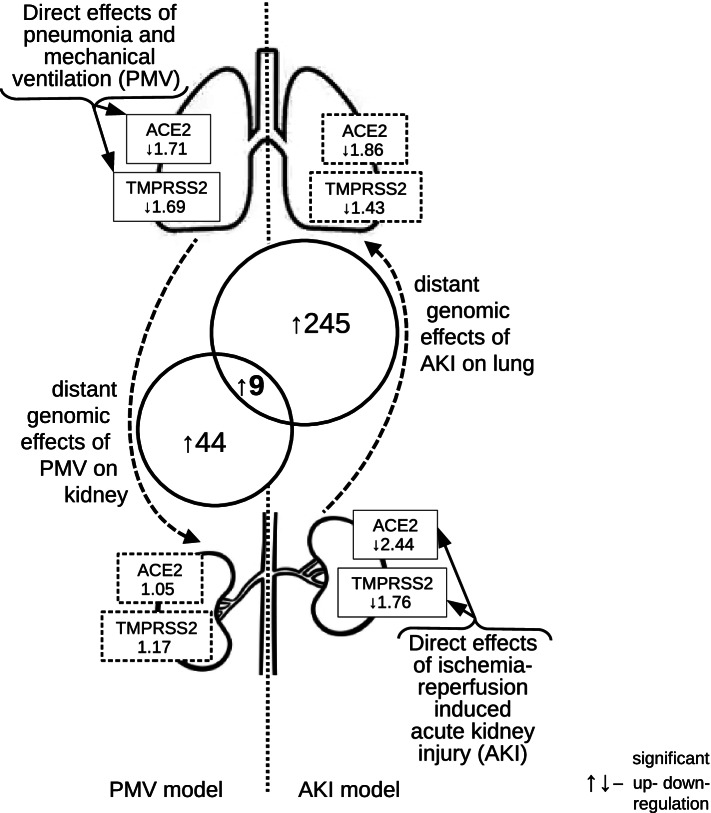
Theoretical model of kidney-lung interaction during COVID-19. We postulate that SARS-CoV2 enters a lung and perhaps a kidney using ACE2 as a main entry point and TMPRSS2 for proteolitic cleavage (priming) of SARS-CoV2-ACE2 complex to increase the infection rate. Ensuing injury (“second hit”), such as ventilation injury and AKI (local tissue inflammation) can alter the expression of these two genes and change response to the infection. The left panel, therefore, represents ventilation injury and right panel represents AKI. Expression values for ACE2 and TMPRSS2 genes are depicted in closed boxes and are taken from Table 1. Solid boxes/lines represent direct effects of an injury, and dashed boxes/lines represent distant genomic effects at 6 h after the injury. Aside from ACE2/TMPRSS2 axis additional pathways can be altered in a lung and kidney that might modify survival and tissue injury/repair. Genes from these key pathways could be involved in lung-kidney cross-talk in COVID-19 patient. We have identified 9 genes, expression of which was significantly changed during experimental PMV and AKI; theses changes were common to both organs during injurious response. Out of common 9 genes 3 were previously described as AKI-related genes (Lcn2, Socs3, and Inhbb); other six genes were novel (Mt2, Maff, Junb, Hmgcs2, Tnfrsf12a and Ch25h)

In kidneys ACE2 sharply decreased at 6 h after AKI and was further downregulated 36 h after the induction of ischemic AKI. However, kidney ACE2 was unaffected by PMV. Kidney TMPRSS2 was unaffected in PMV model, but decreased 6 h after ischemic AKI. (Table 1). We then explored other molecular targets in both organs by filtering for the known genes with *P*-value < 0.01 and fold change (FC) > 4. This approach identified 53 genes upregulated in the kidneys from mice with PMV; and 254 genes upregulated in the lungs from mice with AKI (Fig. 1). Cross-referencing of these candidates identified 9 common genes (Table 2). Three of them Lcn2, Socs3, and Inhbb had already been associated with kidney-lung cross-talk [9], which validate by our current approach. We also identify 6 novel molecular targets for kidney lung-cross-talk.

**Table 2:** Gene candidates distantly upregulated in kidney or lung by injury to distant organ

Gene	Symbol	lung during AKI		kidney in PMV	
		FC	*P* value	FC	*P* value
Lipocalin 2	Lcn2	**18.58**	0.00020	**6.32**	0.00720
Suppressor of cytokine signaling 3	Socs3	**10.52**	0.00018	**10.36**	0.00058
v-maf musculoaponeurotic fibrosarcoma oncogene family F	Maff	**7.21**	0.00221	**5.98**	0.00091
Jun B proto-oncogene	Junb	**6.41**	0.00023	**5.37**	0.00194
Inhibin beta-B	Inhbb	**6.20**	0.00041	**6.17**	0.00102
Tumor necrosis factor receptor superfamily, member 12a	Tnfrsf12a	**6.09**	0.00063	**4.48**	0.00081
Cholesterol 25-hydroxylase	Ch25h	**6.04**	0.00040	**4.30**	0.00037
Metallothionein 2	Mt2	**5.29**	0.00078	**7.09**	0.00003
3-hydroxy-3-methylglutaryl-Coenzyme A synthase 2	Hmgcs2	**4.44**	0.00877	**5.31**	0.00044

*AKI* acute kidney injury, *PMV* pneumonia and mechanical ventilation, *FC* fold change

## Discussion

Severe COVID-19 is usually characterized by lung injury and often associated with acute kidney injury. There has been mechanistic studies of combined lung and kidney injury in animal models, but not related to COVID-19. Prior studies focusing on mechanisms of kidney-lung cross-talk did not examine COVID-19 specific genes because they were not relevant at the time. Publicly available databases can be re-analyzed while labs are slowed down and animal models of COVID-19 are being developed. We used this approach and found unexpected decreases in key SARS-CoV2 molecules ACE2 and TMPRSS2 in two models of kidney lung cross-talk: 1) pneumonia and mechanical ventilation as a trigger of kidney injury and 2) AKI as a trigger of lung injury. Consequently, we found significant changes in expression of 3 genes that were previously associated with kidney-lung cross-talk, and also identified 6 novel targets.

**Fig. 2:**
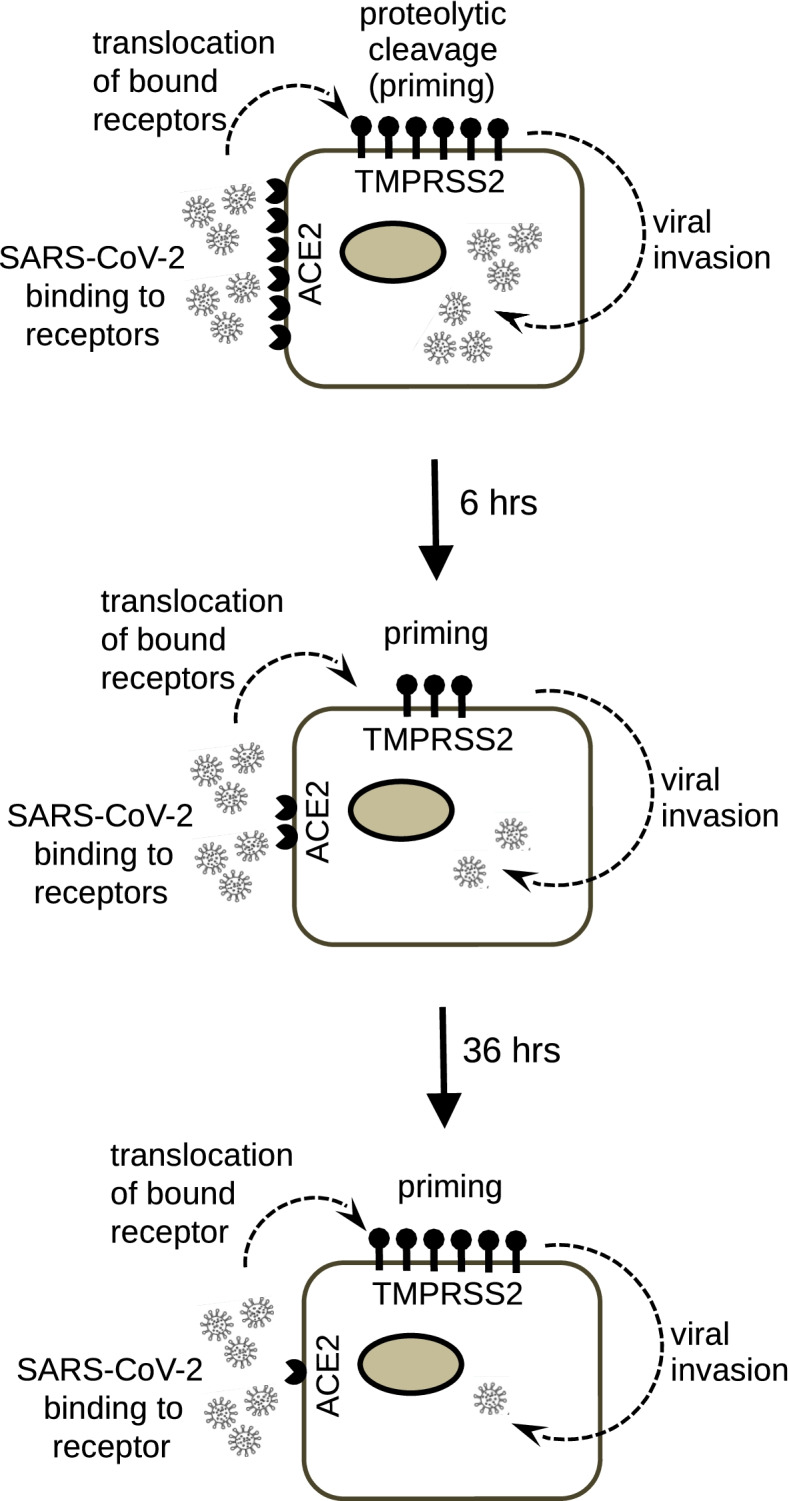
Protective model of a “second hit” during distant organ injury. The schematic cells represent proximal tubule cells of kidney. It has been shown in the Human Protein Atlas that ACE2 and TMPRSS2 are co-expressed in proximal tubules, especially in its microvilli (https://www.proteinatlas.org/ENSG00000130234-ACE2/tissue/kidney and https://www.proteinatlas.org/ENSG00000184012-TMPRSS2/tissue/kidney). The virus invasion flow was adopted from Parmar et al. [12]. The proposed model demonstrates that with increased time after the initial injury it becomes more difficult for SARS-CoV-2 to enter the injured lung or kidney cell. 6 h after the injury the both targets of SARS-CoV-2 (ACE2 and TMPRSS2) are downregulated. 36 h after the injury, while the expression of TMPRSS2 is recovered, the expression of ACE2 is downregulated even more than after 6 h

ACE2 and TMPRSS2 are important for SARS-CoV2 entry into lungs via inhalation and kidneys via blood stream. It is believed that expression level of these molecules predisposes to viral invasion, especially the co-expression of both genes in targeted cells, which significantly accelerates infection and disease development. This could explain why some patients get more serious injury while others do not [16]. There are also “second hits” that can alter patient outcome, such as bacterial pneumonia, mechanical ventilation induced injury, and AKI. We examined expression of these two molecules in two traditional published models and expected an increase in mRNA expression of the receptors. Unexpectedly, both ACE2 and TMPRSS2 decreased in lung and kidney during PMV and AKI, though with different intensities and kinetics. This decrease is a real suppression of expression, rather than a reflection of a cell death after the injury, given that 53 kidney genes and 254 lung genes demonstrated significant increase in response to the injury. The meaning of this is unknown and could be tested in the future studies when experimental models of COVID-19 are better developed. The matter is also complicated by the known difference in expression of TPMRSS2 in the mouse and human [17]. However, one still can speculate that “second hits” could make it more difficult for SARS-CoV-2 to enter lung and kidney cells and might be protective. Based on the level of gene expression in Table 2, we directly extrapolated the gene expression values to receptor expression on the surface of a cell and built our protective model against SARS-CoV-2 invasion (Fig. 2). This model might suggest a straightforward reduction in the severity of COVID-19 infection, which was not noted during this pandemic. However, the future comparison of virus burden between patients with and without AKI can address feasibility of this model.

While focusing on the important COVID-19 targets ACE2 and TMPRSS2 molecules, we re-analyzed these data sets and confirmed that our approach was valid when we identified 3 previously known candidates of lung-kidney cross-talk during AKI: lipocalin 2 (NGAL), suppressor of cytokine signaling 3 (member of STAT family), and inhibin beta B. NGAL is a bio-marker of AKI but also has important role in iron metabolism, epithelial cell and immune cell functions. Socs3 in stressed proximal tubules plays an important role during AKI by inhibition of reparative proliferation [18] and its anti-inflammatory properties might also play protective role in kidney injury. Inhibin beta B is a member of the transforming growth factor β (TGF-β) superfamily. It has been reported that this gene is significantly up-regulated in renal endothelial cells from male mice with ischemia reperfusion injury [19]. We also identified 6 new candidate kidney-lung cross-talk genes (Table 2). We found Maff gene particularly interesting. This gene is abundantly expressed in the kidney and its up-regulation has been linked to deleterious effects on podocytes [20]. Maff also binds the oxytocin receptor promoter [21], which could contribute to sex differences in disease outcome – seen in COVID-19 [22]. Moreover, it had been demonstrated in the highly related mouse model of SARS-CoV that ovariectomised or antiestrogen-treated female mice had more severe CoV infections than their respective controls [23].

## Conclusions

Study of expression patterns of ACE2 and TMPRSS2 genes during PMV and AKI could be important in understanding the pathogenesis of COVID-19 in critically ill patients. These two genes may also interplay with other key genes during kidney-lung cross-talk. Analytical methods of repurposing available genomics data allowed us to generate new COVID-19 related hypotheses, which can be studied and validated once reliable COVID-19 models are established. Moreover, this in silico repurposing method is generalizable and can be applied to different studies.

## Data Availability

The data supporting the results reported in this manuscript can be found at GEO: https://www.ncbi.nlm.nih.gov/geo/query/acc.cgi?acc=GSE60088 https://www.ncbi.nlm.nih.gov/geo/query/acc.cgi?acc=GSE6730

## Abbreviations

ACE2: Angiotensin-converting enzyme 2
AKI: Acute kidney injury
CoV: Corona virus
COVID: Corona virus disease
FC: Fold change
GEO: Gene Expression Omnibus
IRI: Ischemia reperfusion injury
Maff: Musculoaponeurotic fibrosarcoma oncogene family F
NGAL: Neutrophil gelatinase-associated lipocalin
PMV: Pneumonia and mechanical ventilation
SARS: Severe acute respiratory syndrome
Socs3: Suppressor of cytokine signaling 3
TGF-β: Transforming growth factor β
TMPRSS2: Transmembrane protease serine 2

## References

[1] Kow CS, Hasan SS (2020). Possible protective effect of renin-angiotensin system inhibitors in COVID-19 induced acute kidney injury. JASN.

[2] Kellum JA, Nadim MK, Forni LG (2020). Sepsis-associated acute kidney injury: is COVID-19 different?. Kidney Int.

[3] Parmar MS (2021). Acute kidney injury associated with COVID-19-cumulative evidence and rationale supporting against direct kidney injury (infection). Nephrology (Carlton).

[4] Hoke TS, Douglas IS, Klein CL, He Z, Fang W, Thurman JM (2007). Acute renal failure after bilateral nephrectomy is associated with cytokine-mediated pulmonary injury. JASN.

[5] Hassoun HT, Grigoryev DN, Lie ML, Liu M, Cheadle C, Tuder RM (2007). Ischemic acute kidney injury induces a distant organ functional and genomic response distinguishable from bilateral nephrectomy. Am J Physiol Renal Physiol.

[6] Kelly KJ (2003). Distant effects of experimental renal ischemia/reperfusion injury. JASN.

[7] Rabb H, Wang Z, Postler G, Soleimani M (2000). Possible molecular basis for changes in potassium handling in acute renal failure. Am J Kidney Dis.

[8] Zarbock A, Schmolke M, Spieker T, Jurk K, Van Aken H, Singbartl K (2006). Acute uremia but not renal inflammation attenuates aseptic acute lung injury: a critical role for uremic neutrophils. JASN.

[9] Grigoryev DN, Liu M, Hassoun HT, Cheadle C, Barnes KC, Rabb H (2008). The local and systemic inflammatory Transcriptome after acute kidney injury. JASN.

[10] Gu C, Qiao W, Wang L, Li M, Song K. Identification of genes and pathways associated with multiple organ dysfunction syndrome by microarray analysis. Mol Med Report. 2018. 10.3892/mmr.2018.8973. 10.3892/mmr.2018.8973 PMC6059685 29749505

[11] Gharib SA, Mar D, Bomsztyk K, Denisenko O, Dhanireddy S, Liles WC (2016). System-wide mapping of activated circuitry in experimental systemic inflammatory response syndrome. Shock.

[12] Parmar MS (2021). TMPRSS2: an equally important protease as ACE2 in the pathogenicity of SARS-CoV-2 infection. Mayo Clin Proc.

[13] Matsuyama S, Ujike M, Morikawa S, Tashiro M, Taguchi F (2005). Protease-mediated enhancement of severe acute respiratory syndrome coronavirus infection. Proc Natl Acad Sci U S A.

[14] Ni W, Yang X, Yang D, Bao J, Li R, Xiao Y (2020). Role of angiotensin-converting enzyme 2 (ACE2) in COVID-19. Crit Care.

[15] Dudoignon E, Caméléna F, Deniau B, Habay A, Coutrot M, Ressaire Q (2021). Bacterial pneumonia in COVID-19 critically ill patients: a case series. Clin Infect Dis.

[16] Batlle D, Soler MJ, Sparks MA, Hiremath S, South AM, Welling PA (2020). Acute kidney injury in COVID-19: emerging evidence of a distinct pathophysiology. JASN.

[17] Vaarala MH, Porvari KS, Kellokumpu S, Kyllönen AP, Vihko PT (2001). Expression of transmembrane serine protease TMPRSS2 in mouse and human tissues. J Pathol.

[18] Susnik N, Sörensen-Zender I, Rong S, von Vietinghoff S, Lu X, Rubera I (2014). Ablation of proximal tubular suppressor of cytokine signaling 3 enhances tubular cell cycling and modifies macrophage phenotype during acute kidney injury. Kidney Int.

[19] Viñas JL, Porter CJ, Douvris A, Spence M, Gutsol A, Zimpelmann JA (2020). Sex diversity in proximal tubule and endothelial gene expression in mice with ischemic acute kidney injury. Clin Sci.

[20] Okabe M, Motojima M, Miyazaki Y, Pastan I, Yokoo T, Matsusaka T (2019). Global polysome analysis of normal and injured podocytes. Am J Physiol Ren Physio.

[21] Kimura T, Ivell R, Rust W, Mizumoto Y, Ogita K, Kusui C (1999). Molecular cloning of a human MafF homologue, which specifically binds to the oxytocin receptor gene in term myometrium. Biochem Biophys Res Commun.

[22] Jin J-M, Bai P, He W, Wu F, Liu X-F, Han D-M (2020). Gender differences in patients with COVID-19: focus on severity and mortality. Front Public Health.

[23] Channappanavar R, Fett C, Mack M, Ten Eyck PP, Meyerholz DK, Perlman S (2017). Sex-based differences in susceptibility to severe acute respiratory syndrome coronavirus infection. JI.

